# Efficient pulmonary nodules classification using radiomics and different artificial intelligence strategies

**DOI:** 10.1186/s13244-023-01441-6

**Published:** 2023-05-18

**Authors:** Mohamed Saied, Mourad Raafat, Sherif Yehia, Magdy M. Khalil

**Affiliations:** 1 Medical Biophysics, Department of Physics, Faculty of Science, Helwan University, Cairo, Egypt; 2 Department of Mathematics, Faculty of Science, Helwan University, Cairo, Egypt; 3 School of Biotechnology, Badr University in Cairo (BUC) and Department of Physics, Faculty of Science, Helwan University, Cairo, Egypt

**Keywords:** Pulmonary nodules, Malignancy, Deep learning, Machine learning, Lung neoplasms

## Abstract

**Objectives:**

This study aimed to explore and develop artificial intelligence approaches for efficient classification of pulmonary nodules based on CT scans.

**Materials and methods:**

A number of 1007 nodules were obtained from 551 patients of LIDC-IDRI dataset. All nodules were cropped into 64 × 64 PNG images , and preprocessing was carried out to clean the image from surrounding non-nodular structure. In machine learning method, texture Haralick and local binary pattern features were extracted. Four features were selected using principal component analysis (PCA) algorithm before running classifiers. In deep learning, a simple CNN model was constructed and transfer learning was applied using VGG-16 and VGG-19, DenseNet-121 and DenseNet-169 and ResNet as pre-trained models with fine tuning.

**Results:**

In statistical machine learning method, the optimal AUROC was 0.885 ± 0.024 with random forest classifier and the best accuracy was 0.819 ± 0.016 with support vector machine. In deep learning, the best accuracy reached 90.39% with DenseNet-121 model and the best AUROC was 96.0%, 95.39% and 95.69% with simple CNN, VGG-16 and VGG-19, respectively. The best sensitivity reached 90.32% using DenseNet-169 and the best specificity attained was 93.65% when applying the DenseNet-121 and ResNet-152V2.

**Conclusion:**

Deep learning methods with transfer learning showed several benefits over statistical learning in terms of nodule prediction performance and saving efforts and time in training large datasets. SVM and DenseNet-121 showed the best performance when compared with their counterparts. There is still more room for improvement, especially when more data can be trained and lesion volume is represented in 3D.

**Clinical relevance statement:**

Machine learning methods offer unique opportunities and open new venues in clinical diagnosis of lung cancer. The deep learning approach has been more accurate than statistical learning methods. SVM and DenseNet-121 showed superior performance in pulmonary nodule classification.

**Graphical abstract:**

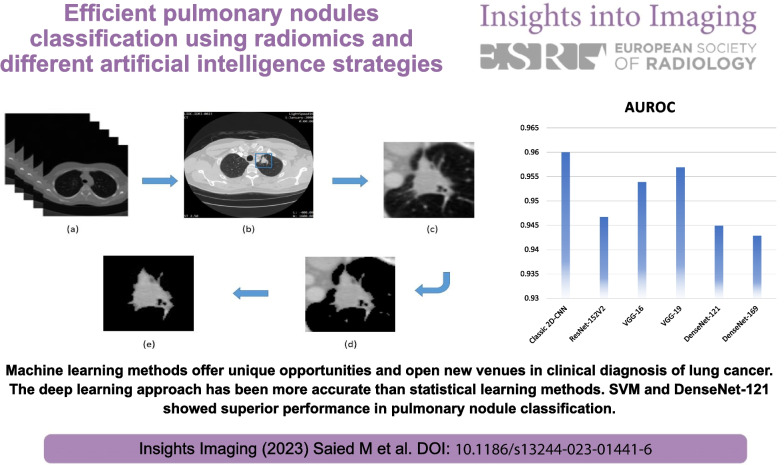

**Supplementary Information:**

The online version contains supplementary material available at 10.1186/s13244-023-01441-6.

## Key Points

1. The deep learning approach has been more accurate than statistical learning methods.
2. SVM and DenseNet-121 showed superior performance in pulmonary nodule classification.
3. The tuning step has been instrumental in improving the classification performance metrics.

**Supplementary Information:**

The online version contains supplementary material available at 10.1186/s13244-023-01441-6.

## Introduction

Lung cancer is the leading cause of cancer death in the USA and around the world [1]. In 2020, there were about more than 2 million new cases diagnosed with lung cancer, accounting for a figure of 11.4% of cancer burden worldwide. In the same year, an estimate of 1.8 million lung cancer deaths was reported [1]. There is an estimate that more than 50% of those who get lung cancer die within 1 year of disease diagnosis [2]. These epidemiological measures emphasize the importance of early pulmonary tumor detection and diagnosis.

Lung cancer begins in the lungs as solitary single nodule or multiple nodules and could spread to lymph nodes or other organs in the body such as brain tissues. Lung tissues are also target for tumor metastases from distant organs.

Computed tomography (CT) image analysis is the gold standard among radiologists in lung cancer diagnosis, which may manifest as lung nodules [3]. These are anomalous globular tissue that can be benign or malignant depending on its characteristics. The issue occurs when lung cancer is in the early stages, which can make it difficult to detect very small tumors. Radiologists are often mentally burdened and overwhelmed to examine many diagnostic images in a single day or session; a reason that may gradually impact his performance over the duty hours [4, 5].

Radiomics aims at enhancing the existing data available to clinicians through application of advanced statistical descriptors and mathematical analysis. Through mathematical extraction of the spatial distribution of signal intensities and pixel interrelationships, radiomics quantifies textural information by using analysis methods from the field of artificial intelligence [6].

The last two decades have witnessed a tremendous increase in artificial intelligence, and many researchers are presently focusing on computer-aided diagnosis. The higher computational abilities of modern computers gave rise to radiomics, a relatively new field that has several features in extracting valuable diagnostic, therapeutic, and prognostic information. It has been demonstrated to have an additional synergistic benefit for assisting medical professionals in making decisions. Hence, combining artificial intelligence/machine learning techniques with radiomics data analysis to classify lung nodules could reduce the need for increasing the number of CT scans, confidently identify lesions, mitigate challenges associated with small lesions, reduce time of the radiologist to diagnose lung nodules resulting in improving accuracy as well as reducing interobserver variability. It can also reduce costs related to diagnostic tests to characterize the disease as well as assisting and/or accelerating data analysis.

The aim of this work was, therefore, to investigate the performance of different artificial intelligence strategies, including statistical learning as well as deep learning methods in the classification of lung nodules being malignant or benign.

## Materials and methods

### Image dataset

The Lung Image Database Consortium image collection (LIDC-IDRI) is a large data repository that contains scanning lung data for the purpose of clinical diagnosis and screening. It is an open-access source for development, hypothesis testing, training, educational purposes and research [7]. It contains 1018 DICOM series of lung CT images that have been fully downloaded from The Cancer Imaging Archive (TCIA) platform [7]. Many cases were noticed to have more than one nodule. Each nodule has been annotated by one or more radiologists and rated from 1 to 5 so that 1 and 2 refer to benign nodules, 4 and 5 refer to malignancy, and the rate of 3 refers to intermediated probability. Among these imaging series, a large number of cases were not included in the reported list of coordinates, and many had single or more nodules annotated with probability rate of 3. In addition, some patients were reported differently without consensus agreement. Because of these issues, only 551 DICOM series of LIDC-IDRI were selected for processing and subsequent analysis. Finally, 1007 nodules have been generated, resulting in 506 lesions reported as benign nodules, whereas 501 were diagnosed malignant.

### Image preprocessing

The aim of data preprocessing is to clean up or remove any probable source of noise and hence provide an improvement of the imaging data. It also serves to suppress undesired distortions or enhances some features for further processing and data analysis.

Previous research efforts were valuable to show different approaches in automatic identification of lung lesions, annotation, segmentation, cropping and feature extraction. In this work, we utilized the successful work implemented by Andrei Teleron with simple modification [4]. Since the lung dataset was annotated in terms of x and y coordinates for the largest 2D slice taken from the axial direction of the lesion volume, the script of Andrei was used to crop lung lesions from selected lung patients. The images obtained were grayscale PNG files of matrix dimension 64 × 64 pixels. The process of cropping the lung lesions has taken approximately 4 min to segment 1007 lung nodules using an average specification laptop computer, Intel(R) Core i5-8250U CPU 3.4 GHz, 4 GB RAM, Intel(R) UHD Graphics 620 supported by NVIDIA GeForce MX 150.

**Fig. 1:**
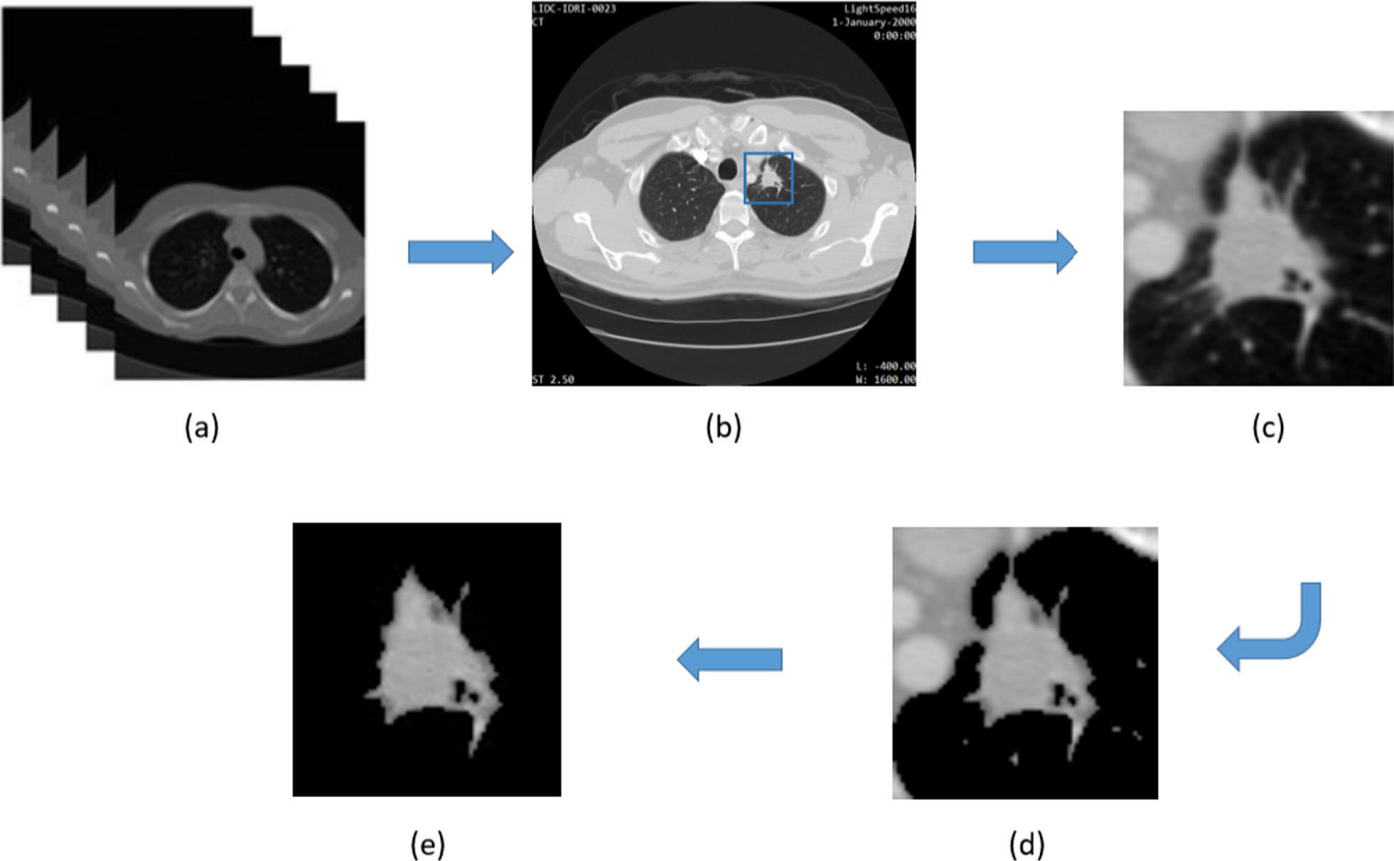
Full preprocessing of lung nodule images. **a** The raw CT imaging data obtained from TCIA and it refers to all CT slices for each patient in 512 × 512 pixels. ** b** Refers to the slice of interest, which was extracted by auto cropping algorithm based on reported annotations of radiologists. **c** Illustrates the cropping process as carried out for the nodules in 64 × 64 image size. **d** Otsu threshold method was used to partially remove non-nodular lung tissues using thresholding. **e** The role of Photo Scissors editor comes to remove manually any remaining non-nodule structure from the images

Attached tissues that weren’t part of the lung nodule were partially removed from all images automatically in few seconds using Otsu thresholding script [8]. Another tool called Photo-Scissors editor, which is an open-source for image editing, was employed to remove manually any remaining non-nodule structure from the images [9]. Pixels that did not contain any structure of the lung nodules were assigned a value of 0 to exclude them from the mask region. The preprocessing steps implemented in this work are illustrated clearly in Fig. 1. The time taken to manually remove extraneous non-nodular tissues varied from one patient to another, being maximum 2 min in the worst case scenario.

### Feature extraction

After image preprocessing, features were extracted from the image and placed in a data frame, each row represents a lung nodule, and each column represents the extracted features. A python script was used for features extraction. This script was able to calculate number of pixels as (nodule size), grey level co-occurrence matrix (GLCM) and local binary pattern (LBP) features.

### GLCM and LBP

GLCM is a statistical method that describes the texture of an image by calculating how often pairs of pixels with certain values and in a specified spatial relationship occur in an image [10], whereas LBP features compute a local representation of texture by comparing each pixel with its neighbors [11].

A total of 18 features were extracted. The first feature was lung nodule size, given as the number of pixels in a single image that had a value above 0. Thirteen textural called Haralick features were computed. These features are based on statistical nature of the most commonly used GLCM [12].

LBP Energy, GLCM Energy, GLCM Homogeneity, GLCM Dissimilarity are additional features that were extracted using “greycomatrix” and “greycoprops”, and LBP histogram functions in skimage library implemented in python [13]. They were then standardized by removing the mean (centers the values from 0 to 1) and scaling to unit variance to reduce bias in the data. The standard python libraries, including Pandas, Numpy, Matplotlib, Skimage, Cv2 and Mahotas, were employed for feature extraction.

### Feature selection

To improve the performance of the model to classify new data and prevent the overfitting issue, the features were reduced from 18 to 4 via one of the most common data reduction techniques. This process was done by using principal component analysis (PCA), which projects the data onto linearly uncorrelated vectors called principle components [14]. It is such that the first component vector has the maximum variance while the subsequent component vector also has the maximum possible variance but constrained in that it is orthogonal to the previous component vector and so on. Once the ranking has been established, the first 4 components were taken, and the rest were discarded. PCA module available in Sklearn python library was used to perform this operation.

### Machine learning methods/classifiers

Machine learning relies on feeding computer algorithms with training data that represent specific patient population in order to build up a set of coefficients/weights in addition to data bias for ultimate model generation. The model is then validated and/or tested for evaluating task performance. The models can be trained for several clinical objectives, including but not limited to image segmentation, detection, classification, staging, and prognosis, and therefore has an important position in patient diagnosis and decision-making paradigm [15].

After preprocessing the CT images into a convenient form with a selection of the significant features to use, the supervised machine learning algorithms were implemented, namely support vector machine (SVM), logistic regression (LR), random forest classifier (RFC), K-nearest neighbor (KNN), Bagging classifier (BC), adaptive boosting (ADB), Bernoulli Naïve Bayes (BNB) and Gaussian Naïve Bayes (GNB), stochastic gradient descent (SGD) and extreme gradient boosting (XGB) were employed. A general description of the major features of those algorithms is mentioned in the Additional file 1.

Stratified k-fold cross-validation was implemented such that data are split/divided into k-folds where each classifier is let to train on K-1 training folds and then used to test on the remaining fold. The testing fold is moved at the end of each iteration until all the data had a chance to be represented in the testing fold [4]. In each iteration, the classifier algorithm was applied, and an accuracy rate was computed. A range of K values were tested (3–10) to see which value resulted in the highest accuracy rate. The value for K was chosen to be 6, and then, the individual values were averaged to obtain a mean accuracy rate for each model. The Sklearn module in Python was used to implement this methodology.

### Deep learning method

Deep learning is an especially complex part of machine learning and considered a universal fitting function. It describes algorithms that look at analyze data with a similar logic pathway similar to human recognition would draw conclusions. Complex, multilayered “deep neural networks” are built to allow data to be passed between nodes (like neurons) in highly connected ways [15]. This enables the processing of unstructured data (images).

### Preparation of input images

First, the images have been split using holdout k-fold into a testing set which included 125 nodules for model evaluation and a training set that included 882 nodules. Images are labeled as (disease: benign/malignant) according to csv file of diagnosis data. Then, a stratified splitting was applied on the training set of 882 nodules, using 80% for actual training and 20% for validation purposes. All sets mentioned are stored in a separated data frame. With Keras Image Data Generator, we could rescale the pixel values and apply random transformation techniques for data augmentation on the fly. We define two different generators. The val_datagen was used to simply rescale the validation and test sets. The train_datagen includes some transformations to augment the train set. Those generators were applied on each dataset using the flow_from_dataframe method. Apart from the transformations defined in each generator, the images are also resized based on the target_size set.

### Classic 2D-CNN building

The different neural layers of the convolutional neural network (CNN) architecture are shown in Table 1. The architecture consists of three blocks where each block includes multiple convolutional layers with rectified linear unit (ReLU) activation function, batch normalization, max-pooling, dropout, flatten and dense layers. At the end, there is a final fully connected sigmoid layer assigned to perform the step of nodule classification, whether benign or malignant. The architecture was tested with RMSprop optimizer. A batch size of 32, 50 epochs, learning rate was set at 0.001, and a binary cross-entropy loss function was used for compiling the model.

**Table 1:** Classic 2D-CNN layers architecture

Layers	Types	Output	Parameters
Input	Input Layer	(None, 64, 64, 3)	0
Block_1	Conv2D_1	(None, 64, 64, 16)	1216
	BatchNormalization_1	(None, 64, 64, 16)	64
	Activation_1 (ReLU)	(None, 64, 64, 16)	0
	MaxPooling2D_1	(None, 32, 32, 16)	0
	Dropout_1	(None, 32, 32, 16)	0
Block_2	Conv2D_2	(None, 32, 32, 32)	4640
	BatchNormalization_2	(None, 32, 32, 32)	128
	Activation_2 (ReLU)	(None, 32, 32, 32)	0
	MaxPooling2D_2	(None, 16, 16, 32)	0
	Dropout_2	(None, 16, 16, 32)	0
Block_3	Conv2D_3	(None, 16, 16, 64)	8256
	Conv2D_4	(None, 16, 16, 64)	16,448
	BatchNormalization_3	(None, 16, 16, 64)	256
	Activation_3 (ReLU)	(None, 16, 16, 64)	0
	MaxPooling2D_3	(None, 8, 8, 64)	0
	Dropout_3	(None, 8, 8, 64)	0
Head	Flatten	(None, 4096)	0
	Dense_1	(None, 64)	262,208
	Dropout_4	(None, 64)	0
Output	Dense_2	(None, 1)	65
	Sigmoid	(None, 1)	0

### Transfer learning using pre-trained model

Transfer learning, as it implies, is the process of transferring previous experience and using/applying it on new data but similar or relevant context. In this step, we used a multiversion of ResNet, VGG-16, VGG-19 and DenseNet as pre-trained models. A description of those neural network models is presented in Additional file 1.

**Fig. 2:**
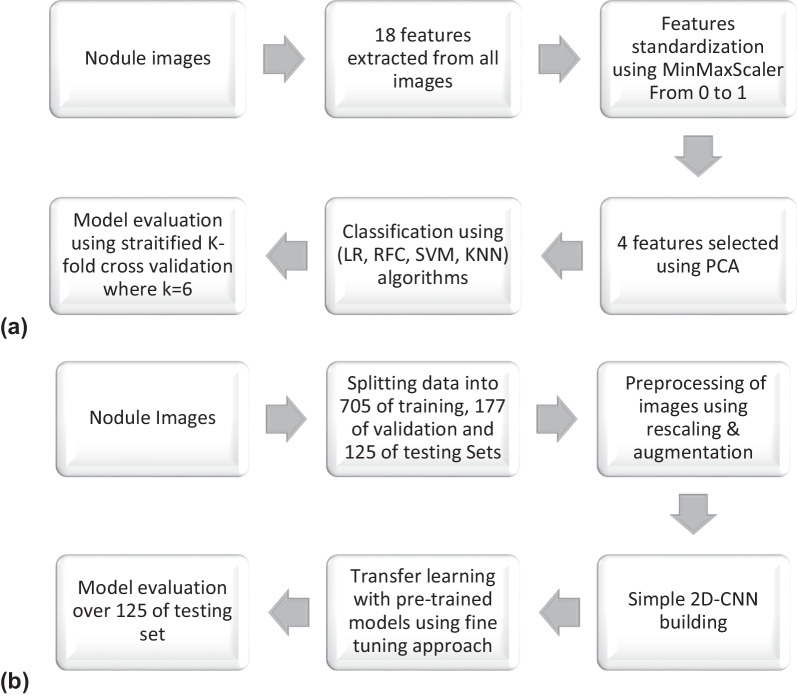
Methods workflow for (**a**) machine learning and (**b**) deep learning

All these models are available on the Keras package and were already trained in another dataset (ImageNet). What we do here is to set include_top to false, removing the ‘head’, responsible for assigning the classes in this other dataset, and keep all the previous layers. Then, we include our last few layers, including the one responsible for generating the output. The layers used as a ‘head’ were global average pooling 2D, dense and dropout. Finally, dense layer with sigmoid function was used as a final layer. Figure 2 illustrates the machine and deep learning workflow.

### Fine tuning

Fine tuning is an approach of model reusability in addition to feature extraction. Specifically, fine tuning is specifically a process that takes a model that has already been trained for one task and then tunes the model to perform a second similar task. It works by unfreezing few of the top layers of the model in neural network used for feature extraction and then combine training both the newly added part of the model (e.g., a fully connected classifier) and the top layers [16].

### Diagnostic performance/model evaluation

Each model has been tested individually over 125 images of testing set, which included 63 benign and 62 malignant nodules, to evaluate all performance metrics for each model.

The diagnostic performance of the different methods and approaches was tested using area under the curve of the receiver operating characteristic analysis, sensitivity, specificity and accuracy [17]. The corresponding formulae of these measures are:

- Sensitivity = TP/(TP + FN)
- Specificity = TN/(TN + FP)
- Accuracy = (TP + TN)/(TP + TN + FP + FN)

where TP is true positive, FN is false negative, TN is true negative, and FP is false positive.

The true positive is a metric used to describe the percent positive that is correctly predicted by the model, whereas true negative is the metric used to describe the percent negative that is correctly diagnosed as negative. In contrast, the false positive is a metric used to describe the percent of incorrectly predicting the positive class, while false negative is the percent of incorrectly detecting the positive nodule as negative.

The area under the curve (AUC) is the measure of the ability of a classifier to distinguish between classes and is used as a summary of the receiver operator characteristic (ROC) curve [18]. The (ROC) curve is an important evaluation/discriminative metric for binary classification problems. It is a probability curve that plots the true positive rate (TPR) against false positive rate (FPR) at various threshold values, where

- TPR = TP/(TP + FN)
- FPR = FP/(FP + TN)

## Results

PCA analysis of the radiomic features resulted in several components such that the first component PC1 did account for 77.7% of the total variance, whereas the second, third and fourth components were 12.5%, 4.1% and 2.2%, respectively. This in total accounts for 96.5% of the total variance.

### Statistical machine learning

**Fig. 3:**
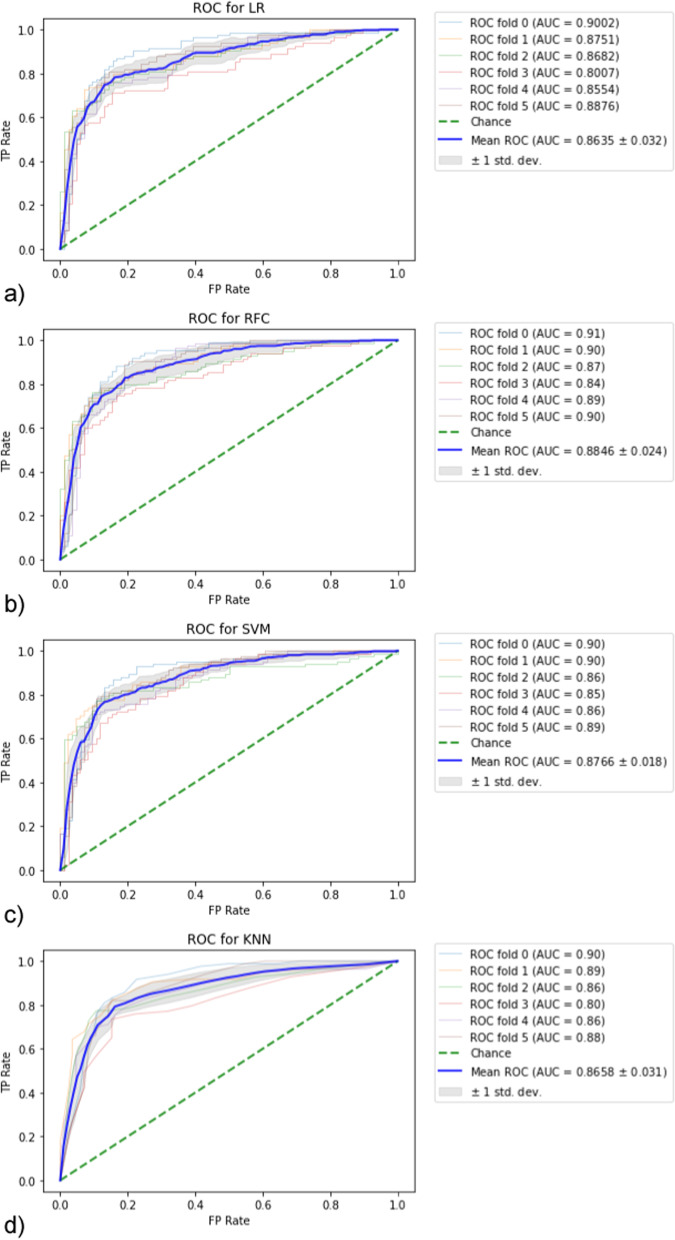

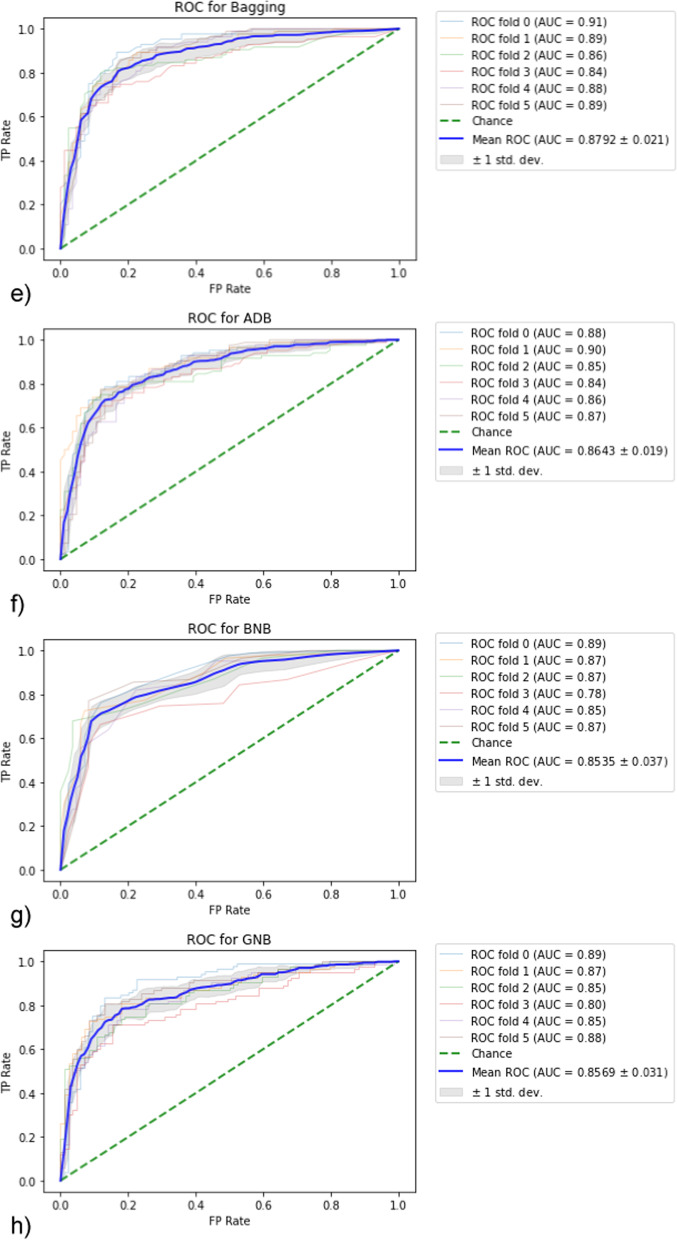

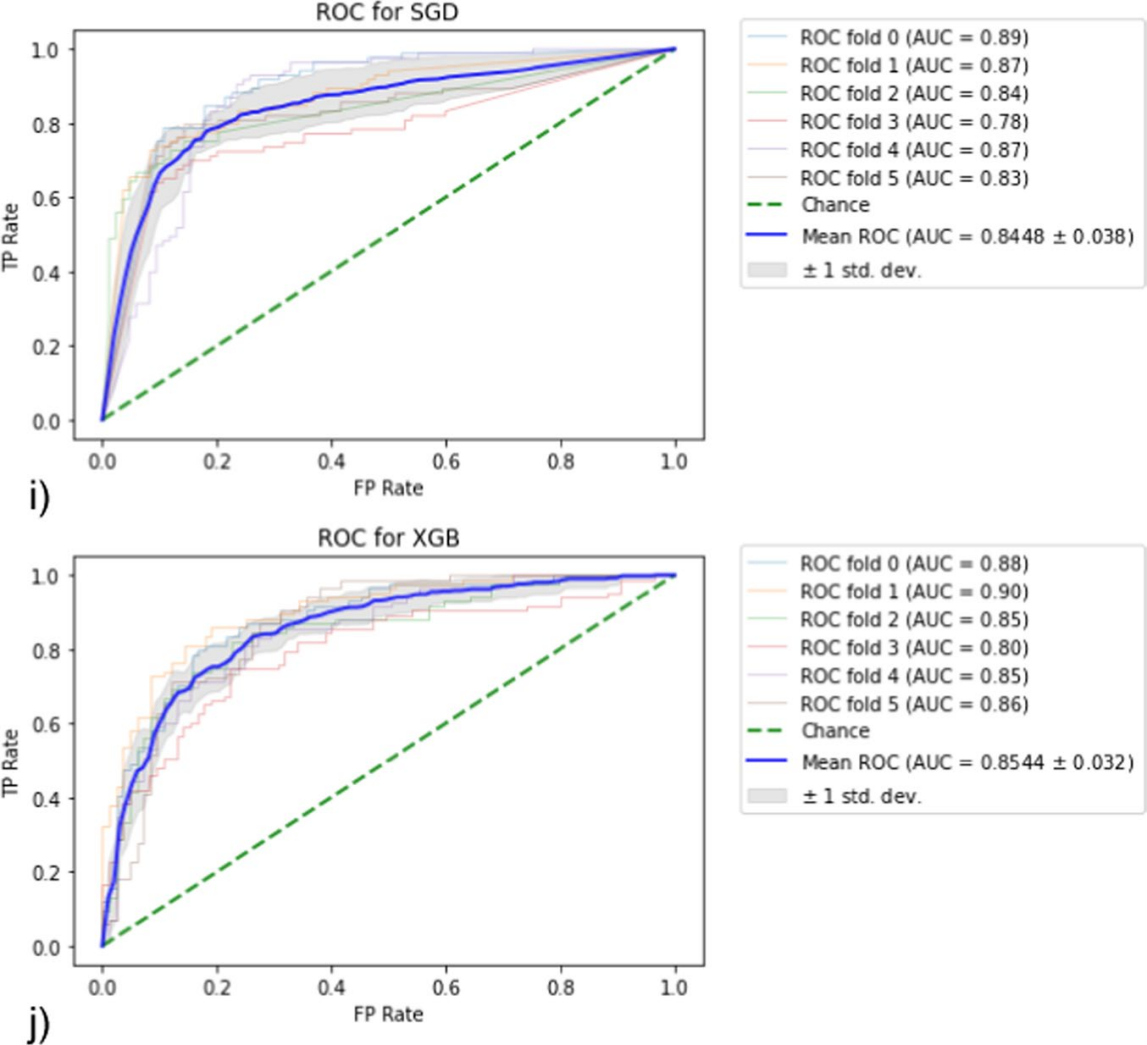
Generated ROC curve following stratified K-fold validation. AUC values for each fold and the resulting mean were calculated. **a** LR, **b** RFC, **c** SVM, **d** KNN, **e** BC, **f** ADB, **g** BNB, **h** GNB, **i** SGD, **j** XGB model

**Fig. 4:**
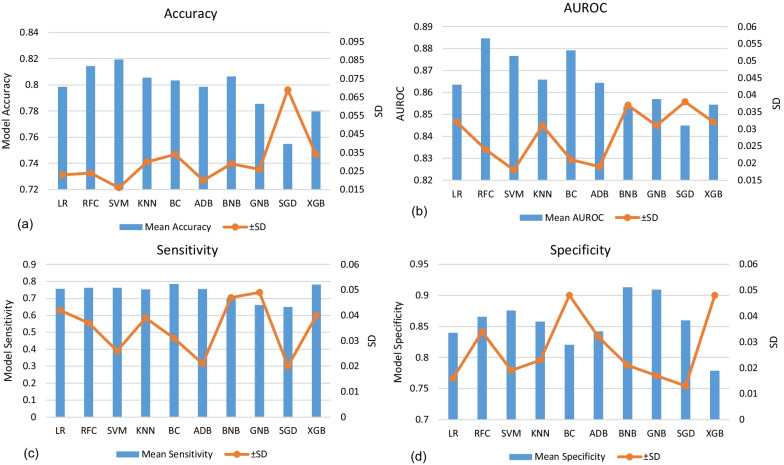
Performance metrics for each model used in statistical learning in classification of pulmonary lung nodules such that each diagram represent the mean of the measured metric calculated for 6-folds along with standard deviation SD plotted on the right vertical axis. **a** model accuracy, **b** model AUROC, **c** model sensitivity and (**d**) the model specificity

In statistical machine learning approach, after running of stratified K-fold cross-validation over all data based on the 10 supervised classifiers, where the number of folds = 6, the generated ROC curves for each model are shown in Fig. 3. Also, the mean accuracy, AUROC, sensitivity and specificity calculated for the 6 folds are demonstrated in Fig. 4.

In terms of models with best measure of sensitivity, the top 3 methods ranked were BC, XGB and SVM, whereas the best 3 methods of test specificity were BNB, GNB and SVM. The best three models in accuracy were SVM, RFC and BNB, whereas those of best measure of ROC were RFC, BC and SVM.

As shown in Fig. 4, the SVM and RFC models provided comparable results in terms of classification accuracy (81.9 ± 1.6% vs. 81.4 ± 2.4%) and sensitivity (76.2 ± 2.6% vs. 76.2 ± 3.7%). Moreover, the RFC was slightly performant in AUROC analysis (88.5 ± 2.4% vs. 87.7 ± 1.8%) but lower than SVM in test specificity (86.6 ± 3.4% vs. 87.6 ± 1.9%).

### Deep and Transfer Learning

**Fig. 5:**
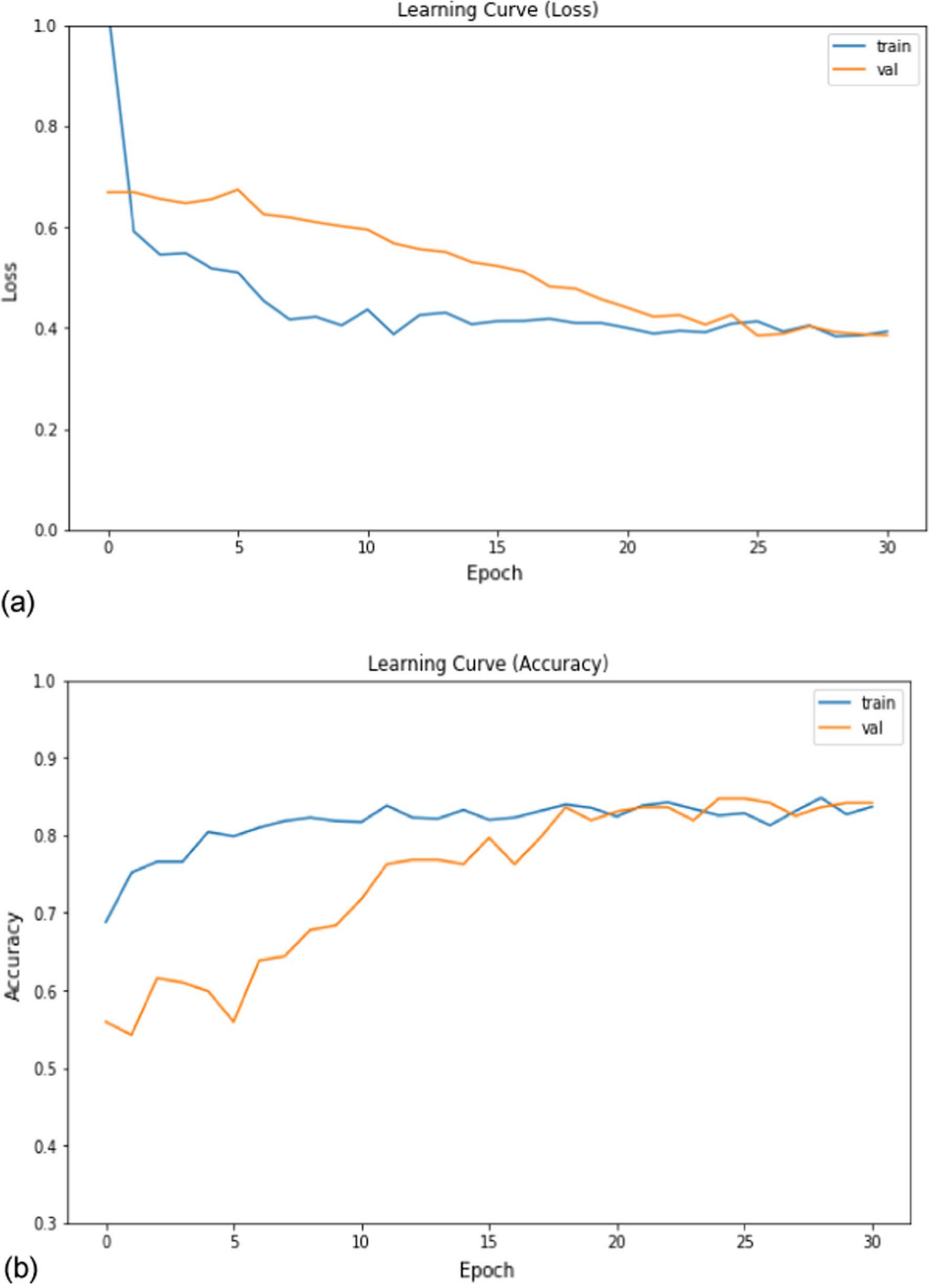
The learning curve for classic 2D CNN model where (**a**) model loss and (**b**) model accuracy

**Fig. 6:**
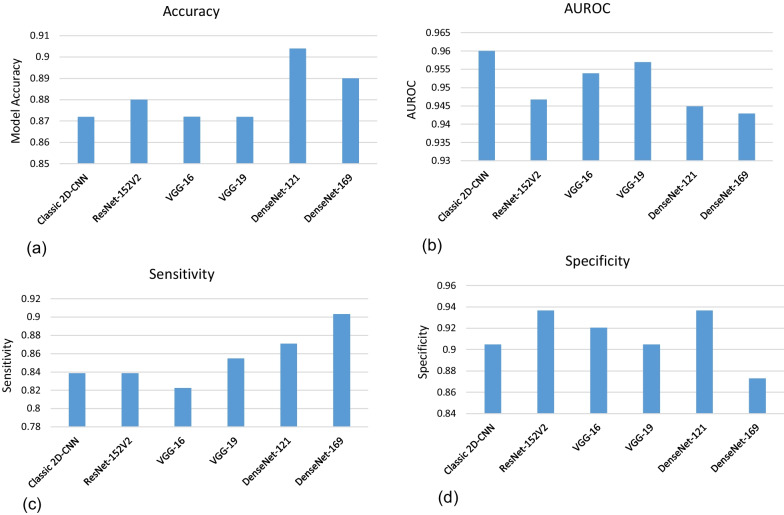
Performance metrics for each model used in transfer deep learning in classification of pulmonary lung nodules. **a** Accuracy, **b** AUROC, **c** sensitivity and (**d**) the specificity

In the deep learning methods, after fitting the classic CNN model over 705 lung nodules of the training set and 177 of the validation set for 50 epochs, the loss and accuracy learning curves were recorded as shown in Fig. 5 to demonstrate how stable the model is. This model has been tested over 125 nodules of testing set, and the performance metrics are summarized in Fig. 6.

With transfer learning and fine tuning approaches, attempts were made to improve the model performance; each pre-trained model has been tested individually using 125 nodules of the testing set, and the results of accuracy, sensitivity, specificity and AUROC are described in Fig. 6. Neural networks that achieved the best accuracy were DenseNet-121 (90.4%) and DenseNet-169 (89.0%) and ResNet-152V2 (88.0%). Data analysis revealed that classic 2D-CNN, VGG-19 and VGG-16 were in order the highest in the AUROC performance analysis (≥ 95%). DenseNet-169, DenseNet-121 and VGG-19 were in order the greatest sensitivity (90.3%, 87.1 and 85.5, respectively) in nodule classification, whereas the best measures of specificity was achieved using DneseNet-121 and ResNet152V2 (both 93.6%) and, afterward, VGG-16 of 92.1%.

## Discussion

Artificial intelligence has become an interesting approach in modern medicine. Computer vision and machine learning, in addition to deep learning approaches to detect, classify as well as predict lung nodule malignancy, have received a particular interest among researchers and practitioners [19]. Several pertinent papers have developed methodologies for estimating the probability of pulmonary nodule malignancy [19]. One study reported an investigation of whether increasing features can improve the performance of classifiers, with emphasis placed on which radiomic features were more useful [20]. The nonlinear classifier yielded an accuracy of 85.7 ± 1.1% and an AUC of 93.2 ± 0.01% using a number of 2817 annotations applying proper data preprocessing and cleaning. When including diameter and volume features, the AUC increased to 0.94 ± 0.01% with an accuracy of 88.1 ± 1.1%.

Another report assembled 5385 valid 3D nodules from 1006 cases derived from LIDC-IDRI database [21]. The singular value decomposition was used in features extraction. Using several popular classifiers, an estimate of classification accuracy of 77.3%, 80.1% and 84.2% was obtained using KNN, random forest and SVM, respectively. In the present work, the same statistical classifiers achieved better accuracy of 80.5%, 81.4% and 81.9%, respectively.

A hybrid approach that integrated GoogLeNet, Denoise Network, and SVM to classify lung nodules is reported, including 742 benign and 553 malignant samples from LIDC-IDRI [22]. The hybrid model achieved an accuracy of 95.0% and AUC of 89.0% [22]. These results are slightly better than the accuracy but lower than the AUC reported here.

One report has adopted transfer learning using three ResNet-50 for classification of lung nodules for identification of lesion appearance, voxel and shape [23]. The accuracy reported using that model for classifying lung nodules was 93.4% [23]. This is better than ResNet-152V2 algorithm employed here, most likely due to the significantly large number of datasets used.

A different approach that includes two deep learning methods, namely AlexNet and ResNet, that were used to extract deep features to classify lung nodules obtained from LUNA 16 dataset [24]. This new architecture was able to achieve accuracy, sensitivity and specificity of 94.3%, 95.5% and 91.1%, respectively. The same model also yielded an ROC of 96.0% and positive predictive value of 89.5% [24]. These results outperform the data presented in this work; however, AlexNet was not used and is worth trying in comparison to other models in future studies.

Comparison of radiomics data and deep learning approaches were conducted on low dose CT dataset extracted from LDCT comprising 1297 lung nodules [25]. The radiomics feature were handcrafted, and deep learning classifiers included were VGG, ResNet, DenseNet, and EfficientNet. The results of the ROC presented here (0.943–0.960) are much better than reported by those transfer learning approaches.

Another recent report has used 1018 cases of LIDC-IDRI dataset and implemented three strategies for classification [26]. They showed that for model modification, the CifarNet performs better than the other modified CNNs with more complex architectures with an ROC of 0.90. Their findings were found consistent with those presented in the present work.

Model sensitivity and specificity are critical performance metrics of a diagnostic test. Improving the detection of truly positive lung cancer patients, especially in early tumor progression, could have a desired outcome as treatment decision would have a strong impact on patient prognosis. This is also apparent in screening or epidemiological studies as early detection is strongly associated with increased prognostic rate. SVM was ranked in the top three approaches in all performance criteria, including sensitivity (81.9%), specificity (87.6%), accuracy (81.9%), and ROC (87.7%), according to the statistical learning methods ranking.

These findings recommend SVM to be an acceptable candidate in designing future algorithms for nodule classification. However, it is inferior to BNB and GNB in specificity measurements, which might question its utility in aggressive treatments (since false positive would be greatly affected) or when strict criteria is placed on the diagnostic test not to include or minimize false positive and/or exclude negative patients. While results obtained for random forest were similar to SVM, it is encouraging to investigate both methods in greater patient population and optimization of hyperparameters.

An accuracy of 94.0% was obtained using modified VGG-16 and transfer learning for solitary lung nodules derived from PET/CT data [27]. The same group also reported model sensitivity, specificity, and AUC of 92.0%, 95.2% and AUC of 93.9%, respectively. A newly developed adaptive boosting self-normalized multiview CNN (ADB-SNMV-CNN) strategy was used in an attempt to improve screening speed, overcome false discovery rate and missed detection [28]. The method was able to achieve an accuracy of 92.0%, sensitivity of 93.0% and specificity of 92.0% with computational rate of 100 min. The ROC was high and slightly greater than the results presented here, 97.6% versus 95.4%, respectively [28]. However, further validation remains to prove the efficacy of the method.

DenseNet-121 was one of the best deep learning classifiers in nodule classification due to its superior performance metrics in accuracy (90.4%), sensitivity (87.1%), specificity (93.6%) and comparable ROC measurements (94.3%) to other methods. The second option is the DenseNet-169 as it showed superior sensitivity and slightly lower accuracy and specificity than DenseNet-121. This drawback of DenseNet-121 is similar to SVM when critical demands are placed on excluding false positive patients.

The current work relied heavily on the TCIA repository having the data annotated and lesion coordination tabulated. This might not be available in some publicly available lung datasets or users who have not an automated or manual annotation. Therefore, in order to integrate our models in a workflow of an integrated pipelines, lung lesion must be initially localized, annotated, extracted, pre-processed and finally prepared as input following a standardized procedure. In future work, automatic localization of the lesion and subsequent data analysis and final decision would be a desired goal to achieve an optimal classification of machine learning algorithms.

The dataset employed in the current study was relatively small, and the classification task was performed on 2D representative images not the whole lesion volume or 3D. The future outlook mandates to tackle this issue besides an increase in data training and testing. External validation from different scanning systems and clinical protocols needs also to be rigorously assessed.

## Conclusions

Deep learning methods were found more accurate and performant than statistical learning methods in classification of lung nodules using CT dataset. In the same context, transfer learning has shown powerful capabilities in reducing efforts and/or time in designing new neural architectures.

The SVM showed competitive performance comparable to the top statistical learning methods in the prediction task. Similarly, the DenseNet-121 was superior deep learning method in lung nodule prediction showing high accuracy, specificity, sensitivity, and comparably high ROC measurements. DenseNet-169 was also superior but with reduced specificity and slightly lower ROC measurements. This to some extent limits the utility of DenseNet-169 and SVM when high specificity is required. The tuning step made in transfer learning has been instrumental in improving the classification metrics. The race of artificial intelligence, including statistical as well as deep learning, would not stop, and several efforts are being made to improve lung cancer diagnosis and treatment.

## Supplementary Information

**Additional file 1.** Brief description of the statistical methods used in lung nodule classifications.

## Data Availability

All data used or analyzed in this study are available from the corresponding author upon reasonable request.

## Abbreviations

AUC = AUROC: Area under receiver characteristic curve
CNN: Convolutional neural network
CT: Computed tomography
FN: False negative
FP: False positive
FPR: False positive rate
GLCM: Grey level co-occurrence matrix
LBP: Local binary pattern
LIDC-IDRI: Lung Image Database Consortium and Image Database Resource Initiative (dataset)
PCA: Principal component analysis
ReLU: Rectified linear unit
ResNet: Residual network
ROC: Receiver operating characteristic (curve)
SVM: Support vector machine
TCIA: The Cancer Imaging Archive
TN: True negative
TP: True positive
TPR: True positive rate
VGG: Visual Geometry Group
