# Supplementary material for: Efficient pulmonary nodules classification using radiomics and different artificial intelligence strategies
Source: Insights Imaging. 2023 May 18;14:91. doi: 10.1186/s13244-023-01441-6 (PMC10195968; doi:10.1186/s13244-023-01441-6)
Supplement: Supplementary file 1 — Additional file 1. Brief description of the statistical methods used in lung nodule classifications. [file 13244_2023_1441_MOESM1_ESM.docx]

**Supplementary Material S1.**

**Statistical learning method**

**Support Vector Machine (SVM)**

In the SVM algorithm, each data item is plotted as a point in n-dimensional space (where n is a number of features) with the value of each feature being the value of a specific coordinate. Then, classification is performed by finding the hyper-plane that differentiates the two classes [1].

**Logistic Regression (LR)**

LR is one of the most famous binary classifiers used in data classifications and predictions. It is an analytic technique that is based on sigmoid function and used to predict the likelihood of a categorical dependent variable [2].

**Random Forest Classifier (RFC)**

RFC creates decision trees based on randomly selected data samples, attain prediction from each tree and selects the best solution by means of voting. It also has the capability to indicate feature importance [3].

**K Nearest Neighbor (KNN)**

The KNN algorithm is a distance based supervised learning classifier and used for classification problems. It is a nonparametric supervised learning classifier that is based on an individual data grouping. The k values is normally taken as the square root of the number of records in the training set. Different methods are used in distance measurements including Euclidean distance, cosine similarity measure, chi-square and others [4].

**Bagging Classifier (BC)**

Bagging is a type of ensemble machine learning approach that combines the outputs from many learners to improve performance. It fits base classifiers each on random subsets of the original dataset and then aggregate their individual predictions either by voting or averaging to give a final prediction [5].

**Adaptive Boosting (ADB)**

An adaptive boosting classifier is a meta-estimator that starts by fitting a classifier on the original dataset and fits additional copies of the classifier on the same dataset but where the weights of incorrectly classified instances are adjusted such that subsequent classifiers focus more on difficult cases [6].

**Bernoulli Naïve Bayes (BNB) and Gaussian Naïve Bayes (GNB)**

BNB and GNB are classification algorithms of machine learning based on Bayes theorem which give the likelihood of occurrence of the event. Bernoulli is used for discrete data and it works on Bernoulli distribution, whereas Gaussian is used for continuous data and work on normal distribution [7].

**Stochastic Gradient Descent (SGD)**

The SGD method is an iterative method for optimizing an objective function with suitable smoothness properties. Gradient descent is one of the most popular algorithms to perform optimization and it can be used as classifier when used to optimize the linear classifiers such as SVM or LR [8].

**Extreme Gradient Boosting (XGB)**

XGB is powerful enough to find any nonlinear relationship between labels and features and has several benefits in dealing with missing values, outliers, and categorization of features without any special treatment. XGB is an open-source library that provides an effective and efficient implementation of the gradient boosting algorithm for classification or regression problems. When the classifiers decision trees based are fit using differentiable loss function and gradient descent optimization, this technique is called gradient boosting [9]. Because the performance of a model significantly depends on the value of hyper-parameters, GridSearch-CV was used with some classifiers to determine the optimal values for the given model [10].

**Supplementary Material S2. Deep learning method**

**ResNeT**

A Residual Neural Network (ResNet) is an artificial neural network that combines residual blocks on top of each other to form the network architecture. This model was selected as the winner of ImageNet challenge in 2015. The fundamental breakthrough with ResNet was it allowed us to train extremely deep neural networks with 150+layers successfully. ResNet is a method that deals with the vanishing gradient problem in very deep CNNs. It works by skipping some layers assuming the fact that very deep networks should not produce a training error greater than its shallow ones. The ResNet has different versions as models used in this study (ResNet-152V2) [11].

**VGG**

VGG architecture is an abbreviation for Visual Geometry Group. The VGG framework is a standard CNN with multiple layers. It forms the basis of ground-breaking object recognition models. The number that follow VGG is the number of deep layers and hence VGG-16 and VGG-19 denotes 16 and 19 computational layers respectively [12].

**DenseNet**

A DenseNet is a type of convolutional neural network that employs dense connections between layers, through dense blocks, where all layers are connected directly with each other. DenseNet is similar to ResNet with some basic variations. The former concatenates the previous layer with the future one whereas the latter uses an additive method that merges both layers. The DenseNet has different versions, like DenseNet-121, DenseNet-169, DenseNet-201, etc. [13].

**References**

[1] Understanding Support Vector Machine(SVM) algorithm from examples (along with code). <https://www.analyticsvidhya.com/blog/2017/09/understaing-support-vector-machine-example-code/>.

[2] Building an End-to-End Logistic Regression Model. <https://www.analyticsvidhya.com/blog/2021/10/building-an-end-to-end-logistic-regression-model/>.

[3] Understanding Random Forests Classifiers in Python Tutorial. <https://www.datacamp.com/tutorial/random-forests-classifier-python>.

[4] Learn all about the K-Nearest Neighbor (KNN) Algorithm in Machine Learning. <https://courses.analyticsvidhya.com/courses/K-Nearest-Neighbors-KNN-Algorithm?utm_source=blog&utm_medium=knn_in_python&R>.

[5] ML | Bagging classifier. <https://www.geeksforgeeks.org/ml-bagging-classifier/>.

[6] An AdaBoost classifier. <https://scikit-learn.org/stable/modules/generated/sklearn.ensemble.AdaBoostClassifier.html>.

[7] Naive Bayes Classifiers. <https://www.geeksforgeeks.org/naive-bayes-classifiers/>.

[8] Introduction to SGD Classifier. <https://michael-fuchs-python.netlify.app/2019/11/11/introduction-to-sgd-classifier/>.

[9] Extreme Gradient Boosting (XGBoost) Ensemble in Python. <https://machinelearningmastery.com/extreme-gradient-boosting-ensemble-in-python/>.

[10] Tune Hyperparameters with GridSearchCV Rahul Shah — Published On June 23, 2021 and Last Modified On July 20th, 2022. <https://www.analyticsvidhya.com/blog/2021/06/tune-hyperparameters-with-gridsearchcv/>.

[11] Deep Residual Networks (ResNet, ResNet50) – 2022 Guide, Gaudenz Boesch,
 <https://viso.ai/deep-learning/resnet-residual-neural-network/>.

[12] VGG Very Deep Convolutional Networks (VGGNet), Gaudenz Boesch, <https://viso.ai/deep-learning/vgg-very-deep-convolutional-networks/>.

[13] Introduction to DenseNets (Dense CNN), [Shivam Baldha](https://www.analyticsvidhya.com/blog/author/shivambaldha/)**,** <https://www.analyticsvidhya.com/blog/2022/03/introduction-to-densenets-dense-cnn/>.
